# Predictors of suicidal ideation in UK doctors: retrospective case–control study from NHS Practitioner Health

**DOI:** 10.1192/bjo.2025.10894

**Published:** 2025-11-25

**Authors:** Ashvin Kuri, Aleksandra Nowak, Andrea Allen-Tejerina, Caitlin Norris-Grey, Kiran Anya Chilu Kuri, Jack Barton, Zaid Al-Najjar, Bhathika Perera, Helen Garr, Jonathan Round

**Affiliations:** Wolfson Institute of Population Health, Queen Mary University of London, UK; St George’s University of London, UK; East & North Hertfordshire NHS Trust, Stevenage, UK; Independent Researcher, Boston, USA; Queen Mary University of London, UK; Auckland City Hospital, Auckland, New Zealand; NHS Practitioner Health, London, UK; Division of Psychiatry, University College London, UK

**Keywords:** Suicide, mental health services, prevention, prognostic/prediction modelling, multivariate statistics

## Abstract

**Background:**

Depression severity is a well-established risk factor for suicidal ideation, but the extent to which sociodemographic and employment-related factors contribute independently remains unclear.

**Aims:**

Complete data from doctors (*N* = 4055) presenting to National Health Service Practitioner Health (NHS-PH) in 2022–2023 were used to test the hypothesis that depression severity is the largest determinant of suicide ideation risk (defined by Patient Health Questionnaire 9 (PHQ-9) question 9 score) among doctors.

**Method:**

Using PHQ-8 score (PHQ-9, excluding the item on suicide ideation) as a proxy for depression severity, the case–control discriminatory capacity of receiver operating characteristic curves (AUCs) were evaluated for (a) a univariable model studying modified PHQ-9 alone as the predictor of severe suicide ideation; and (b) a multivariable model integrating modified PHQ-9 and multiple sociodemographic and employment factors as the predictor of severe suicide ideation. Models were compared both descriptively and through a likelihood ratio test.

**Results:**

The univariable model using depression severity alone as the predictor of severe suicide ideation yielded an AUC of 0.921. The addition of sociodemographic and employment factors improved the fit significantly (likelihood ratio test with (*χ* ^2^(14) = 50.26, *P* < 0.001), amended AUC 0.930). Having both a disability and a relationship status of ‘no partner’ was significantly independently associated with suicide ideation in the multivariable model.

**Conclusions:**

In this national cohort of doctors, depression severity was strongly associated with suicidal ideation. However, disability and lack of a partner were also independently linked to increased risk, suggesting that suicidal ideation is not solely driven by symptom severity. Social and functional factors may help identify higher-risk individuals and inform targeted support.

Mental and emotional difficulties among doctors are common, yet chronically underreported.^1–3^ Suicidal ideation and attempts by doctors to take their lives represent the most severe form of such difficulties. Drivers of suicide ideation in doctors have been studied quantitatively, qualitatively and theoretically. The interplay of sociodemographic factors (age, gender, disability, ethnicity, being a carer, relationship status, non-UK medical school, anxiety levels, depression severity) and employment factors (work pattern, specialty, support at work) are thought to coalesce in susceptible individuals to drive thoughts of self-harm and suicide.^1–3^

However, exploring suicide ideation among doctors is difficult, and even estimating prevalence is remarkably challenging. The reasons for this are fourfold. First, because doctors present to multiple services for support with mental health difficulties, centralising presentations into a single database is challenging. Second, because doctors’ mental illness is a highly sensitive area, research is subject to particular ethical scrutiny. Third, there will be many who have suicidal ideation but do not seek support, nor report ideation to any service.^4^ Finally, study ‘outcomes’ are difficult to compare. For those who go on to complete suicide, it is rarely possible objectively to explore their mental state before this event. Even using suicidal ideation as an outcome in a study is confounded by heterogeneous metrics across instruments and databases. For instance, how suicide ideation is captured by two commonly used scales – Patient Health Questionnaire 9 (PHQ-9)^5^ and Clinical Outcomes in Routine Evaluation-10 (CORE-10)^6^ – is different, and this may also differ from an individual clinical assessment.^7^

The difficulty in exploring the area is exemplified by the wide variation in quantitative estimates of suicidal prevalence. The relative risk of doctor suicide compared with the general population has been reported as low as 0.67 and as high as 2.55, and is notably more common in female doctors.^8^ Depression is considerably more common in doctors than in the general public,^3^ while doctors have a lower prevalence of most physical health conditions. The consensus is that this ‘healthy worker effect’ does not hold true for depression, suicide ideation and suicide itself.^9^

While suicide and suicidal ideation share nomenclature, their relationship is more nuanced; prediction and intervention remain inherently challenging. Nevertheless, the existing evidence seems to suggest that a significant proportion of those who complete suicide had some preceding prodrome of suicidal ideation.^10,11^ Restated, there is compelling evidence that suicide ideation is a strong risk factor for completing suicide. A better understanding of such individuals with suicidal ideation is therefore necessary, and may provide a target for interventions to reduce rates of completed suicide among doctors. While depression is a core risk factor for suicide ideation,^12^ contemporary theoretical frameworks – including the interpersonal theory of suicide, the integrated motivational–volitional model and ideation-to-action models – emphasise that suicide ideation emerges from a combination of affective distress and psychosocial or contextual stressors, such as social isolation, disability or perceived entrapment.^13^ These models imply that suicide ideation may not simply be a marker of more severe depression, but rather a distinct psychological state shaped by specific social and functional risks.

Previous studies of suicidality in doctors have been limited by small sample sizes, lack of structured assessments or non-clinical populations. Our study uses a large UK cohort to explore two competing hypotheses: (a) that suicide ideation is a direct function of depression severity (i.e. a symptom of more extreme affective disturbance), versus (b) that suicide ideation reflects a distinct risk state in which sociodemographic and occupational factors contribute independently to risk.

## Method

### National Health Service Practitioner Health

National Health Service Practitioner Health (NHS-PH) is a confidential service in the UK dedicated to mental health disorders among healthcare workers, with doctors being the largest professional group accessing the service. Since 2008, NHS-PH has seen over 35 000 clients.^14^ Healthcare staff with a wide range of difficulties are encouraged to self-refer and are often signposted by their employer, family doctor or occupational health services. Practitioner Health provides NHS-funded care through a multidisciplinary team including mental health nurses, general practitioners, psychiatrists and psychotherapists, all specialising in the treatment of healthcare professionals.

NHS-PH is a self-referral service, with most patients registering online via a simple web-based form, eliminating the need for third-party referrals. For those who encounter difficulties with the form, registration can also be completed by telephone. Once submitted, each registration is reviewed by an experienced clinician and allocated to an appropriate assessment and treatment pathway. Patients are then usually able to self-select a convenient date and time for their initial consultation.

This paper describes a retrospective case–control study of anonymised data reported via NHS-PH between 2022 and 2023. Where appropriate, the Strengthening the Reporting of Observational Studies in Epidemiology (STROBE) guidelines were followed.^15^

### Data collection, processing and choice of primary measure

Anonymised data were supplied to the research team by NHS-PH as above. Data comprised sociodemographic information and the mental health scores PHQ-9 (appendix 1 in the supplementary material available at https://doi.org/10.1192/bjo.2025.10894) and Generalised Anxiety Disorder Score 7 (GAD-7, appendix 2), as well as a range of anonymised details related to the participants’ current employment. This was collected during the initial booking-in process (as above). PHQ-9 and GAD-7 are widely accepted and validated screening questionnaires used in population-based mental health research and clinical practice, and have been validated internationally. Data represented all doctors from any clinical specialty presenting to NHS-PH who consented to their data being used for research, from 2022 to 2023. Records were excluded if data were incomplete.

### Cohorts

From the overall data-set, a ‘case’ cohort of doctors with frequent suicidal ideation was delineated (*n* = 206). Cases were defined as doctors who reported suicide ideation ‘nearly every day over the last 2 weeks’, as defined by a PHQ-9 question 9 (PHQ-9 Q9) score of 3 points (maximum) (appendix 1). This stringent cut-off was considered to represent the most unequivocal definition of suicide ideation from the data available.^16^

**Fig. 1:**
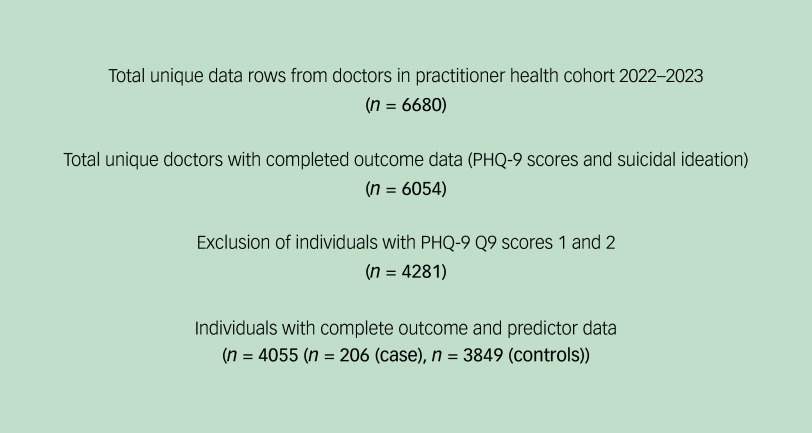
Flowchart of patient inclusion criteria. PHQ-9, Patient Health Questionnaire 9; PHQ-9 Q9, PHQ-9 question 9.

The controls in fitted models were defined as those with a PHQ-9 Q9 score of 0 (minimum), i.e. zero thoughts of being ‘better off dead, or thoughts of hurting [one]self in some way’ over the past 2 weeks. We excluded individuals with a PHQ-9 Q9 score of 1 (‘several days’) due to ambiguity in clinical interpretation. While Q9 = 1 may indicate fleeting or passive suicidal thoughts, it lacks the frequency or severity often associated with clinically actionable ideation. Including these responses risks heterogeneity in the case group and may dilute associations. Those scoring 2 (‘more than half the days’) were also excluded, to preserve a clear separation between cases (frequent ideation, Q9 = 3) and controls (no ideation, Q9 = 0). This approach prioritises internal validity and allows more confident interpretation of predictors in the extremes – recognising that a binary outcome simplifies the underlying continuum – but yields a more clinically meaningful contrast for exploratory modelling. This yielded a final cohort of cases (*n* = 206) and controls (*n* = 3849) (Fig. 1).

### Statistical analysis

To evaluate whether factors beyond depression severity improved the prediction of suicidal ideation (suicide ideation), we compared two logistic regression models: a base model including only the PHQ-8 score as a measure of depression severity, and a full model incorporating additional sociodemographic and clinical variables (including age, gender, ethnicity, disability status, relationship status and GAD-7 score). Model performance was assessed using two metrics. First, we calculated the area under the receiver operating characteristic curve (AUC) for each model to quantify its discriminatory ability. Second, we performed a likelihood ratio test to determine whether the inclusion of additional covariates significantly improved model fit beyond PHQ-8 alone. These complementary approaches allowed us to assess both the predictive utility and statistical value of incorporating non-depression factors into risk modelling.

Both the uni- and multivariable models had a good model fit, as assessed by Hosmer and Lemeshow goodness of fit (*P* > 0.05). The multivariable model had a statistical absence of collinearity (as measured by variance inflation factor – all <1.5).

Finally, we examined the case–control discriminatory performance of the brief PHQ-2 (first two questions of the PHQ-9 questionnaire) in a univariable (PHQ-2) univariate (suicidal ideation) logistic regression model. We compared the performance of this univariable (PHQ-2) model with that of the univariable (PHQ-8) model through Delong’s test (AUC comparison), and by comparison of Akaike information criterion (AICs). These findings may help to inform minimum assessment requirements for future research involving suicidal risk and ideation.

All analysis was conducted in R using RStudio (version 2024.04 for macOS; RStudio, Integrated Development Environment for R; Posit Software, PBC, Boston, Massachusetts, USA; http://www.posit.co/).

## Results

A total of 6680 individuals consented for their data to be used for research purposes, of whom 6054 (90.6%) had complete data. With a cohort that is predominantly female, working full-time and non-disabled, and with a higher proportion of non-White doctors, the cohort appears to be representative of the underlying doctor workforce in the UK (Table 1).

**Table 1:** Descriptive statistics of variables included in multivariable model

Factor	Cases	Controls
	Categorical/binary, *n* (%)	Categorical/binary, *n* (%)
Gender		
Female	139 (67.5)	2788 (72.4)
Male	67 (32.5)	1061 (27.6)
Partner status		
Has partner	97 (47.1)	2842 (73.8)
No partner	109 (52.9)	1007 (26.2)
Carer status		
Yes	73 (35.4)	1802 (46.8)
No	133 (64.6)	2047 (53.2)
Full-time (FT) versus less than full-time (LTFT)		
FT	133 (64.6)	2103 (54.6)
LTFT	73 (35.4)	1746 (45.4)
Disability		
Yes	61 (29.6)	465 (12.1)
No	145 (70.4)	3384 (87.9)
Overseas-trained doctor		
Yes	53 (25.7)	820 (21.3)
No	153 (74.3)	3029 (78.7)
Being under Greater Medical Council or local investigation		
Yes	28 (13.6)	299 (7.8)
No	178 (86.4)	3550 (92.2)
Ethnicity (binary)		
Yes	92 (44.7)	1414 (36.7)
No	114 (55.3)	2435 (63.3)
Seeking help with substance abuse		
Yes	16 (7.8)	113 (2.9)
No	190 (92.2)	3736 (97.1)
Has medical comorbidities		
Yes	112 (54.4)	1753 (45.5)
No	94 (45.6)	2096 (54.5)
Working status		
Working	118 (57.3)	2866 (74.5)
Sick leave	72 (35.0)	761 (19.8)
Other	16 (7.8)	222 (5.8)

	Continuous, mean (± s.d.)	Continuous, mean (± s.d.)
Age (years)	39.88 (±9.77)	40.52 (±9.09)
Alcohol (units per week)	6.47 (±22.36)	5.44 (±9.91)
GAD-7 score	16.24 (±4.34)	11.18 (±5.24)
PHQ-8 score	20.22 (±3.58)	10.85 (±5.29)

GAD-7, Generalised Anxiety Disorder Score 7; PHQ-8, Patient Health Questionnaire 8.

### Uni- and multivariable model characteristics

The univariable model (PHQ-8 alone) yielded an AUC of 0.921, demonstrating excellent case–control discriminatory ability. The addition of sociodemographic and clinical covariates to the model led to a statistically significant improvement in fit (likelihood ratio test, *χ* ^2^(14) = 50.26, *P* < 0.001), with an increase in AUC to 0.930.

### Factors associated with suicidal ideation in the multivariable model

We demonstrate that being disabled (odds ratio 1.81, 95% CI: 1.18–2.78, *P* = 0.0065), having a relationship status of ‘no partner’ (odds ratio 2.41, 95% CI: 1.67–3.49, *P* < 0.00005) and higher PHQ-8 score (odds ratio 1.53 per 1-point increase in PHQ-8 score, 95% CI: 1.45–1.62, *P* < 0.00005) are significantly associated with suicide ideation. The full set of results from the multivariable model is presented in Table 2.

**Table 2:** Factors associated with suicidal ideation in multivariable model

Factor	Odds ratio	CI	*P*-value
PHQ-8 score	1.53 (per 1-point increase)	1.45–1.62	**<0.00005**
Disability (Yes)	1.81	1.18–2.78	**0.0065**
Relationship status of ‘No partner’ (compared with having a partner)	2.41	1.67–3.49	**<0.00005**
Working full-time	1.32	0.89–1.95	>0.05
Age (years)	0.99 (per 1-year increase)	0.97–1.01	>0.05
Being a carer	0.98	0.66–1.47	>0.05
Being an overseas-trained doctor	0.83	0.54–1.25	>0.05
Being under local or GMC investigation	1.74	0.95–3.08	>0.05
Male gender (versus female)	1.03	0.69–1.51	>0.05
Non-White ethnicity	0.83	0.58–1.20	>0.05
Seeing help with substance abuse	1.44	0.68–2.92	>0.05
Having a medical comorbidity	0.84	0.57–1.21	>0.05
Being on sick leave (versus not working for other reasons)	0.82	0.39–1.80	>0.05
Currently employed (versus not working for other reasons, excluding sick leave)	0.80	0.39–1.72	>0.05
GAD-7 score	1.00 (per 1-point increase in score)	0.96–1.05	>0.05

GAD-7, Generalised Anxiety Score 7; GMC, Greater Medical Council; PHQ-8, Patient Health Questionnaire 8.

Bold are significant factors (*p* < 0.05).

### PHQ-2 performance in a univariable univariate model

PHQ-2 exhibits significant case–control differentiation, with an AUC of 0.912. This is not statistically significantly different to the AUC of the PHQ-8 model (0.921), as assessed using DeLong’s test (*P* = 0.22). However, using a different metric of comparison (comparing AICs: PHQ-2 = 1047 *v*. PHQ-8 = 1007, ΔAIC = 40) suggests that the PHQ-8 model probably does provide a better fit.

## Discussion

We present evidence from this case–control study within the NHS-PH database to suggest that depression severity (odds ratio 1.53 per 1-point increase in PHQ-8 score, 95% CI: 1.45–1.62, *P* < 0.00005) is the biggest determinant of suicide ideation among doctors presenting with mental health difficulties in the UK. However, the addition of sociodemographic and employment factors made the model significantly better at identifying suicide ideation than using PHQ-8 alone, based on a likelihood ratio test (*χ* ^2^(14) = 50.26, *P* < 0.001). We also demonstrate that, among UK doctors, being disabled (odds ratio 1.81) and having a relationship status of ‘no partner’ (odds ratio 2.41) are independently associated with suicidal ideation.

It is well evidenced that being disabled as a doctor presents a range of additional workplace challenges that could plausibly negatively influence an individual’s mental health. Although disability is a known risk factor for suicide and suicidal ideation among the general population,^10,17^ this has been less explored among doctors. Where cohort studies have examined ‘suicide-related behaviours’ (of which suicide ideation is one) among disabled doctors, they tend to report a significant association. For example, a cohort study of physicians with autism spectrum disorder demonstrated a higher risk of suicide attempts.^18^ In our study, we provide evidence of the strong association between disability and suicide ideation among UK doctors, but unpicking potential causal reasons for this association is complex. A starting point is the 2024 British Medical Association (BMA, UK) report, which highlighted that doctors with disabilities frequently felt unsupported by their workplaces and peers, struggled with obtaining the reasonable adjustments necessary to function optimally in the workplace and felt pressured to return to work before being ready.^11^ Potentially, suicide ideation may be a sequela of these workplace difficulties.

Meaningful positive relationships are well explored as protective factors against suicidal ideation, although findings remain mixed.^19^ For example, studies from Norway have suggested that ‘single status’ is associated with increased risk of suicide ideation.^20^ Further granularity from this Norwegian cohort suggests that the absence of cohabitation may be a key intermediate, or causal, variable explaining this association.^21^ However, other studies have reported null findings with respect to relationship/marital status and suicide ideation, such as a large 2022 Japanese study.^22^ In this study, we report that having a status of ‘no partner’ is significantly associated with increased risk of suicide ideation. In this cohort ‘no partner’ included widowers and divorcees, and it is therefore difficult to unpick whether it is actually these stressful life events driving the observed signal, rather than just the fact that one is single.

Interestingly, previous research consistently demonstrates elevated suicide risk among female physicians internationally.^23–26^ However, the absence of gender differences in suicidal ideation in our physician-only cohort suggests that this vulnerability may manifest through pathways not captured by suicidal ideation measures, or that women physicians experiencing suicidal thoughts face higher barriers to help-seeking and thus would not have been in our cohort.

Our finding that the univariable model using PHQ-2 provides a similar model fit to the univariable model using PHQ-8 suggests that PHQ-2 is an easily accessible, and brief, screening tool that may have utility in future research projects related to suicide and suicidal ideation. We note that the significant difference in AIC (Δ40 points) suggests that, if researchers want maximal explanatory power, PHQ-8 is better than PHQ-2 and, of course, that the multivariable model integrating sociodemographic and employment factors is statistically significantly better than PHQ-8 itself.

The limitations of this study must be recognised. While Practitioner Health does represent a large, reputable and recognised organisation where doctors may seek support for their mental health, it will not capture every doctor with suicidal ideation in the UK. Some with suicidal ideation will present to their own primary care practitioners, and some will not seek help. In the nuanced field of doctors’ mental health, it is also somewhat reductive to reduce the variables in the study to categorical, and to ignore the nuance of the nature of variables such as ‘being disabled’, ‘being a carer’ and having ‘no partner’. It is very challenging to capture such nuance while retaining sufficient power for analysis. It is also important to consider the potential for reverse causality in the model, such as suicide ideation leading to relationship breakdown and worsening of other features of depression, thus being potentially confounding. However, importantly, even if causality cannot be concretely established, these factors may still serve as valuable predictors for identification of at-risk individuals and targeting of interventions. The study also relied on self-reported data, rather than researcher-assessed data, which is important to consider as a limitation. It is also important to recognise that this study was conducted within a UK population only, and this limits its generalisability to other healthcare workforces where different sociocultural and country-specific employment factors may be at play. Finally, it is important to consider the risk of selection bias in this cohort because these were voluntarily consenting participants. No data were captured on participants who did not consent to the use of their data in research, and this may be a meaningful cohort that is absent from analysis.

Our research identifies several areas for future work. Research on cohorts with deeper granularity into variable subcategories (disability, partner status, nature of disciplinary action) would yield more precise conclusions. Replicating our findings across different cohorts, both within the UK and abroad, is another important next step. Finally, exploring our models longitudinally with future longitudinal data from Practitioner Health, with a particular emphasis on completed suicide as an outcome, is an important area of future research.

In conclusion, this study shows that depression severity, disability and not having a partner are strongly associated with suicide ideation among UK doctors. These factors may help identify individuals at higher risk and could be targets for support. Tailored interventions for doctors with disabilities or without partner support, alongside appropriate psychological care for depression, may help reduce the burden of suicide ideation in this population.

## Supporting information

Kuri et al. supplementary material Kuri et al. supplementary material

## Data Availability

Data from National Health Service Practitioner Health (NHS-PH) are available to academic researchers by direct application to Practitioner Health. Applications are evaluated based on scientific rigour and require a clear research question and proposal. Separately, data dictionaries are available and can be provided through contacting Practitioner Health directly.

## Supplementary material

The supplementary material is available online at https://doi.org/10.1192/bjo.2025.10894
