# Supplementary material for: Predictors of suicidal ideation in UK doctors: retrospective case–control study from NHS Practitioner Health
Source: BJPsych Open. 2025 Nov 25;11(6):e285. doi: 10.1192/bjo.2025.10894 (PMC12724124; doi:10.1192/bjo.2025.10894)
Supplement: Kuri et al. supplementary material [file S2056472425108946sup001.docx]

**Appendix 1**

**PHQ-9 — Patient Health Questionnaire - 9-item**

| Over the last 2 weeks, how often have you been bothered by any of the following problems? | Not at all | Several Days | More than half the days | Nearly every day |
| --- | --- | --- | --- | --- |
| 1. Little interest or pleasure in doing things | 0 | 1 | 2 | 3 |
| 1. Feeling down, depressed, or hopeless | 0 | 1 | 2 | 3 |
| 1. Trouble falling asleep, staying asleep, or sleeping too much | 0 | 1 | 2 | 3 |
| 1. Feeling tired or having little energy | 0 | 1 | 2 | 3 |
| 1. Poor appetite or overeating | 0 | 1 | 2 | 3 |
| 1. Feeling bad about yourself, feeling that you are a failure, or feeling that you have let yourself or your family down | 0 | 1 | 2 | 3 |
| 1. Trouble concentrating on things such as reading the newspaper or watching television | 0 | 1 | 2 | 3 |
| 1. Moving or speaking so slowly that other people could have noticed. Or being so fidgety or restless that you have been moving around a lot more than usual | 0 | 1 | 2 | 3 |
| 1. Thinking that you would be better off dead or that you want to hurt yourself in some way | 0 | 1 | 2 | 3 |

| If you checked off any problem on this questionnaire so far, how difficult have these  problems made it for you to do your work, take care of things at home, or get along  with other people? | | | |
| --- | --- | --- | --- |
| Not difficult at all | Somewhat difficult | Very difficult | Extremely difficult |

**Appendix 2**

**GAD-7 — Generalized Anxiety Disorder 7-item**

| Over the last 2 weeks, how often have you been bothered by any of the following problems? | Not at all | Several Days | More than half the days | Nearly every day |
| --- | --- | --- | --- | --- |
| 1. Feeling nervous, anxious, or on edge | 0 | 1 | 2 | 3 |
| 1. Not being able to stop or control worrying | 0 | 1 | 2 | 3 |
| 1. Worrying too much about different things | 0 | 1 | 2 | 3 |
| 1. Trouble relaxing | 0 | 1 | 2 | 3 |
| 1. Being so restless that it is hard to stay still | 0 | 1 | 2 | 3 |
| 1. Becoming easily annoyed or irritable | 0 | 1 | 2 | 3 |
| 1. Feeling afraid, as if something awful might happen | 0 | 1 | 2 | 3 |

| If you checked any problems, how difficult have they made it for you to do your work, take care of  things at home, or get along with other people? | | | |
| --- | --- | --- | --- |
| Not difficult at all | Somewhat difficult | Very difficult | Extremely difficult |

**Appendix 3**

**CORE-10 — Clinical Outcomes in Routine Evaluation 10-item**

| Over the last week… | Not at all | Only occasionally | Some  times | Often | Most or all of the time |
| --- | --- | --- | --- | --- | --- |
| 1. I have felt tense, anxious or nervous | 0 | 1 | 2 | 3 | 4 |
| 1. I have felt I have someone to turn to for support when needed | 4 | 3 | 2 | 1 | 0 |
| 1. I have felt able to cope when things go wrong | 4 | 3 | 2 | 1 | 0 |
| 1. Talking to people has felt too much for me | 0 | 1 | 2 | 3 | 4 |
| 1. I have felt panic or terror | 0 | 1 | 2 | 3 | 4 |
| 1. I made plans to end my life | 0 | 1 | 2 | 3 | 4 |
| 1. I have had difficulty getting to sleep or staying asleep | 0 | 1 | 2 | 3 | 4 |
| 1. I have felt despairing or hopelessness | 0 | 1 | 2 | 3 | 4 |
| 1. I have felt unhappy | 0 | 1 | 2 | 3 | 4 |
| 1. Unwanted images or memories have been distressing me | 0 | 1 | 2 | 3 | 4 |

**Appendix 4**

**STROBE Statement**

|  | **Item No.** | **Recommendation** | **Page  No.** | **Relevant text from manuscript** |
| --- | --- | --- | --- | --- |
| **Title and abstract** | 1 | (*a*) Indicate the study’s design with a commonly used term in the title or the abstract | 1 | A case-control study exploring suicidal ideation in a large cohort of UK doctors presenting with mental health difficulties |
|  |  | (*b*) Provide in the abstract an informative and balanced summary of what was done and what was found | 2 | **Methods**: Complete data from doctors (n=4055) presenting to NHS Practitioner Health (NHS-PH) in 2022-23 was used to test the hypothesis that depression severity was the largest determinant of risk of SI amongst doctors. Using the PHQ8 score (PHQ9 excluding the item on SI) as a proxy for depression severity, the case-control discriminatory capacity of receiver operating characteristic curves (ROC) were evaluated for 1) a univariable model studying the modified PHQ9 alone as the predictor of severe SI; 2) a multivariable model integrating modified PHQ9 and multiple sociodemographic and employment factors, as the predictor of severe SI. Models were compared descriptively, and through a likelihood ratio test.  **Findings**: The univariable model using depression severity (PHQ8) alone as the predictor of severe SI yielded an area under the curve (AUC) of 0.921. The addition of sociodemographic and employment factors improved fit significantly (Likelihood ratio test with (χ²(14) = 50.26, *p*<0.001), new AUC 0.930). Having a disability and having a relationship status of ‘no partner’ were significantly independently associated with SI in the multivariable model. |
| **Introduction** | | | |  |
| **Background / rationale** | 2 | Explain the scientific background and rationale for the investigation being reported | 3-4 | Mental and emotional difficulties amongst doctors are common, yet chronically underreported (1–3).  Drivers of suicidal ideation in doctors have been studied quantitatively, qualitatively, and theoretically. The interplay of sociodemographic factors (age, gender, disability, ethnicity, being a carer, relationship status, non-native medical school, anxiety levels, depression severity) and employment factors (work pattern, specialty, support at work), are thought to coalesce in susceptible individuals to drive thoughts of self-harm and suicide.    However, exploring suicidal ideation amongst doctors is difficult.  For those who go on to complete suicide, it is rarely possible to objectively explore their mindset before this event. Even using suicidal ideation as an ‘outcome’ in a study is confounded by heterogeneous metrics across instruments and databases.    There is compelling evidence that suicidal ideation is a strong risk factor for completing suicide. A better understanding of such individuals with suicidal ideation is therefore necessary, and may provide a target for interventions to reduce rates of completed suicide amongst doctors. |
| **Objectives** | 3 | State specific objectives, including any prespecified hypotheses | 4 | To understand the prevalence and risk factors of suicidal ideation amongst UK doctors.  To quantitatively identify factors associated with suicidal ideation amongst UK doctors. |
| **Methods** | | | | |
| **Study design** | 4 | Present key elements of study design early in the paper | 5 | Retrospective case-control study of anonymised data reported via NHS Practitioner Health (NHS-PH) between 2022–2023. |
| **Setting** | 5 | Describe the setting, locations, and relevant dates, including periods of recruitment, exposure, follow-up, and data collection | 5 | NHS-PH is a confidential service dedicated to mental health disorders amongst healthcare workers, with doctors being the largest professional group accessing the service. Since 2008, NHS-PH has seen over 35,000 clients (12).    Anonymised data was supplied to the research team by NHS-PH as above. Data comprised socio-demographic information, the mental health scores PHQ9 (appendix 1) and Generalised Anxiety Disorder Score 7 (GAD-7, appendix 2) as well as a range of anonymised details related to participants’ current employment. This was collected during the initial booking-in process. PHQ9 and GAD-7 are widely accepted and validated screening questionnaires used in population-based mental health research and clinical practice. Data represented all doctors from any clinical specialty presenting to NHS-PH who consented to their data being used for research, from 2022-23. Records were excluded if data were incomplete. 6,054 records with complete data in this period were available for retrospective analysis (of 6,680 total records). |
| **Participants** | 6 | (*a*) *Cohort study*—Give the eligibility criteria, and the sources and methods of selection of participants. Describe methods of follow-up  *Case-control study*—Give the eligibility criteria, and the sources and methods of case ascertainment and control selection. Give the rationale for the choice of cases and controls  *Cross-sectional study*—Give the eligibility criteria, and the sources and methods of selection of participants | 5 | From the overall dataset, a ‘case’ cohort of doctors with frequent suicidal ideation was delineated (n = 206). Cases were defined as doctors who reported SI ‘nearly every day over the last two weeks’, as defined by PHQ-9 Question 9 score of 3 points (maximum) (appendix 1). This stringent cut-off was felt to represent the most unequivocal definition of SI from the data available (13).  The controls in fitted models were defined as those with a PHQ-9 Question 9 score of 0 (minimum), i.e., zero thoughts of being ‘better off dead, or thoughts of hurting [one]self in some way’ over the last two weeks. We excluded individuals with a PHQ-9 Q9 score of 1 (‘several days’) due to ambiguity in clinical interpretation. While Q9=1 may indicate fleeting or passive suicidal thoughts, it lacks the frequency or severity often associated with clinically actionable ideation. Including these responses risks heterogeneity in the case group and may dilute associations. Those scoring 2 (‘more than half the days’) were also excluded to preserve a clear separation between cases (frequent ideation, Q9=3) and controls (no ideation, Q9=0). This approach prioritises internal validity and allows more confident interpretation of predictors in the extremes — recognising that a binary outcome simplifies the underlying continuum, but yields a more clinically meaningful contrast for exploratory modelling. This yielded a final cohort of cases (n = 206) and controls (n = 3849). |
|  |  | (*b*) *Cohort study*—For matched studies, give matching criteria and number of exposed and unexposed  *Case-control study*—For matched studies, give matching criteria and the number of controls per case | 5 | From the overall dataset, a ‘case’ cohort of doctors with frequent suicidal ideation was delineated (n = 206). Cases were defined as doctors who reported SI ‘nearly every day over the last two weeks’, as defined by PHQ-9 Question 9 score of 3 points (maximum) (appendix 1). This stringent cut-off was felt to represent the most unequivocal definition of SI from the data available (13).  The controls in fitted models were defined as those with a PHQ-9 Question 9 score of 0 (minimum), i.e., zero thoughts of being ‘better off dead, or thoughts of hurting [one]self in some way’ over the last two weeks. We excluded individuals with a PHQ-9 Q9 score of 1 (‘several days’) due to ambiguity in clinical interpretation. While Q9=1 may indicate fleeting or passive suicidal thoughts, it lacks the frequency or severity often associated with clinically actionable ideation. Including these responses risks heterogeneity in the case group and may dilute associations. Those scoring 2 (‘more than half the days’) were also excluded to preserve a clear separation between cases (frequent ideation, Q9=3) and controls (no ideation, Q9=0). This approach prioritises internal validity and allows more confident interpretation of predictors in the extremes — recognising that a binary outcome simplifies the underlying continuum, but yields a more clinically meaningful contrast for exploratory modelling. This yielded a final cohort of cases (n = 206) and controls (n = 3849). |
| **Variables** | 7 | Clearly define all outcomes, exposures, predictors, potential confounders, and effect modifiers. Give diagnostic criteria, if applicable | 6 | To evaluate whether factors beyond depression severity improved the prediction of suicidal ideation (SI), we compared two logistic regression models: a base model including only the PHQ-8 score as a measure of depression severity, and a full model incorporating additional sociodemographic and clinical variables (including age, gender, ethnicity, disability status, relationship status, and GAD-7 score). Model performance was assessed using two metrics. First, we calculated the area under the receiver operating characteristic curve (AUC) for each model to quantify their discriminatory ability. Second, we performed a likelihood ratio test to determine whether the inclusion of additional covariates significantly improved model fit beyond PHQ-8 alone. These complementary approaches allowed us to assess both the predictive utility and statistical value of incorporating non-depression factors into risk modelling. |
| **Data sources / measurement** | 8* | For each variable of interest, give sources of data and details of methods of assessment (measurement). Describe comparability of assessment methods if there is more than one group |  | Anonymised data was supplied by NHS Practitioner Health (NHS-PH), collected during the initial booking-in process.  Data comprised socio-demographic information, the mental health scores PHQ9 (appendix 1) and Generalised Anxiety Disorder Score 7 (GAD-7, appendix 2) as well as a range of anonymised details related to participants’ current employment.  PHQ9 and GAD-7 are widely accepted and validated screening questionnaires used in population-based mental health research and clinical practice. |
| **Bias** | 9 | Describe any efforts to address potential sources of bias | 10-11 | The limitations of this study must be recognised. Whilst Practitioner Health does represent a large, reputable, and recognised organisation for doctors to seek support for their mental health, it will not capture every doctor with suicidal ideation in the UK. Some with suicidal ideation will present to their own primary care practitioners, and some will not seek help. In the nuanced field of doctor’s mental health, it is also somewhat reductive to reduce the variables in the study to categorical, and ignore the nuance of the nature of variables such as ‘being disabled’, ‘being a carer’, and having ‘no partner’. It is very challenging to capture such nuance whilst retaining sufficient power for analysis. It is also important to consider the potential for reverse causality in the model, such as SI leading to relationship breakdown and worsening other features of depression, thus being potentially confounding. The study also relied on self-reported data, rather than researcher-assessed data, which is important to consider as a limitation. Finally, it is important to consider the risk of selection bias in this cohort as these are voluntarily consenting participants. No data is captured on participants who did not consent to the use of their data in research, and this may be a meaningful cohort that is absent from analysis. |
| **Study size** | 10 | Explain how the study size was arrived at | 5-6 | Figure 1. |

| **Quantitative Variables** | 11 | Explain how quantitative variables were handled in the analyses. If applicable, describe which groupings were chosen and why | 5, 7-8 | PHQ-8, GAD-7, and age were treated as continuous variables. Other variables were binary (e.g. disability: yes/no) or categorical (e.g. working status). Category definitions provided in Table 2. |
| --- | --- | --- | --- | --- |
| **Statistical methods** | 12 | (*a*) Describe all statistical methods, including those used to control for confounding |  | Logistic regression was used to model the outcome (SI) with multiple covariates included to adjust for confounding.  Model diagnostics included ROC AUC and likelihood ratio testing. |
|  |  | (*b*) Describe any methods used to examine subgroups and interactions |  | N/A |
|  |  | (*c*) Explain how missing data were addressed | 5 | Records were excluded if data were incomplete. |
|  |  | (*d*) *Cohort study*—If applicable, explain how loss to follow-up was addressed  *Case-control study*—If applicable, explain how matching of cases and controls was addressed  *Cross-sectional study*—If applicable, describe analytical methods taking account of sampling strategy | 6 | Adjustment for covariates in multivariable models. |
|  |  | (*e*) Describe any sensitivity analyses | 6 | Two separate multivariable models. Variables were included based on the evidence base. |
| **Results** | | | | |
| **Participants** | **13*** | (a) Report numbers of individuals at each stage of study—eg numbers potentially eligible, examined for eligibility, confirmed eligible, included in the study, completing follow-up, and analysed | 6 | Total unique data rows from doctors in Practitioner Health Cohort 2022-23  (n=6,680)    Total unique doctors with complete outcome data (PHQ9 scores and suicidal ideation)  (n=6,054)    Exclusion of individuals with PHQ9 Q9 scores 1 and 2  (n = 4281)    Individuals with complete outcome data, and complete predictor data  (n=4,055) |
|  |  | (b) Give reasons for non-participation at each stage | 6 | Records excluded due to incomplete data. Participants with PHQ-9 Q9 = 1 or 2 excluded due to ambiguous SI status. See details in methods. |
|  |  | (c) Consider use of a flow diagram | 6 | Please see Figure 1. |
| **Descriptive data** | **14*** | (a) Give characteristics of study participants (eg demographic, clinical, social) and information on exposures and potential confounders | 8-9 | Descriptive statistics of participants presented for both cases and controls across all predictor variables (see Table 1). |
|  |  | (b) Indicate number of participants with missing data for each variable of interest | 6 | All participants included in analysis had complete data for all model variables; incomplete records were excluded. |
|  |  | (c) *Cohort study*—Summarise follow-up time (eg, average and total amount) | 1 | This is a retrospective case-control study with no follow-up. |
| **Outcome data** | **15*** | *Cohort study*—Report numbers of outcome events or summary measures over time |  |  |
|  |  | *Case-control study—*Report numbers in each exposure category, or summary measures of exposure | 8-9 | Primary outcome: Suicidal ideation defined by PHQ-9 Q9 = 3 (cases) vs. Q9 = 0 (controls)  Number of outcome events (cases): 206  Number of controls: 3849  Table 2 reports:  - ORs, 95% CIs, p-values for all predictors  - PHQ-8 score (continuous exposure)  - Other exposures: disability, relationship status, etc.  Table 1 shows stratified counts for each predictor variable, by outcome group |
|  |  | *Cross-sectional study—*Report numbers of outcome events or summary measures |  |  |
| **Main results** | **16** | (*a*) Give unadjusted estimates and, if applicable, confounder-adjusted estimates and their precision (eg, 95% confidence interval). Make clear which confounders were adjusted for and why they were included | 7-9 | Adjusted ORs with 95% CI and p-values reported in Table 1. Model adjusted for PHQ-8 and full set of sociodemographic and employment covariates as described in Methods. |
|  |  | (*b*) Report category boundaries when continuous variables were categorized | 7-9 | No continuous variables (e.g., PHQ-8, GAD-7, age) were categorised in the regression model. Binary variables are defined in Table 1 (e.g. disability: yes/no; relationship status: partner/no partner). Case-control outcome defined by PHQ-9 Q9 = 3 vs 0. |
|  |  | (*c*) If relevant, consider translating estimates of relative risk into absolute risk for a meaningful time period |  | Not applicable – case-control design with no underlying incidence data. Absolute risk cannot be calculated. |

| **Other analyses** | **17** | Report other analyses done—eg analyses of subgroups and interactions, and sensitivity analyses | 7 | Univariable and multivariable model performance compared using AUC and likelihood ratio test (Figure 1).  No subgroup or interaction analyses were performed. |
| --- | --- | --- | --- | --- |
| **Discussion** | | | | |
| **Key results** | **18** | Summarise key results with reference to study objectives | 10 | Depression severity (OR 1.53 per 1-point increase in PHQ8 score, 95% CI 1.45-1.62, p<0.00005) is the biggest determinant of SI amongst doctors presenting with mental health difficulties in the UK.  The addition of a comprehensive range of sociodemographic and employment factors significantly improved model fit in a multivariable model, as assessed through a likelihood ratio test (χ²(14) = 50.26, p<0.001).  We also demonstrate being disabled (OR 1.81), and having a relationship status of ‘no partner’ (OR 2.41), are independently associated with suicidal ideation amongst UK doctors. |
| **Limitations** | **19** | Discuss limitations of the study, taking into account sources of potential bias or imprecision. Discuss both direction and magnitude of any potential bias | 5-6 | The limitations of this study must be recognised. Whilst Practitioner Health does represent a large, reputable, and recognised organisation for doctors to seek support for their mental health, it will not capture every doctor with suicidal ideation in the UK. Some with suicidal ideation will present to their own primary care practitioners, and some will not seek help. In the nuanced field of doctor’s mental health, it is also somewhat reductive to reduce the variables in the study to categorical, and ignore the nuance of the nature of variables such as ‘being disabled’, ‘being a carer’, and having ‘no partner’. It is very challenging to capture such nuance whilst retaining sufficient power for analysis. It is also important to consider the potential for reverse causality in the model, such as SI leading to relationship breakdown and worsening other features of depression, thus being potentially confounding. The study also relied on self-reported data, rather than researcher-assessed data, which is important to consider as a limitation. Finally, it is important to consider the risk of selection bias in this cohort as these are voluntarily consenting participants. No data is captured on participants who did not consent to the use of their data in research, and this may be a meaningful cohort that is absent from analysis. |
| **Interpretation** | **20** | Give a cautious overall interpretation of results considering objectives, limitations, multiplicity of analyses, results from similar studies, and other relevant evidence | 1 | Depression severity is the largest determinant of SI in this large cohort of doctors presenting to NHS-PH.  The increased risk of SI in those who are disabled and those who have ‘no partner’ may, in context, help risk stratify doctors presenting with mental health difficulties. |
| **Generalisability** | **21** | Discuss the generalisability (external validity) of the study results | 5-6 | Participants were UK doctors presenting to NHS-PH who consented to research use and had complete data.  Results are most generalisable to doctors in similar support settings with clearly defined high or low SI.  Generalisability is limited to those who sought help via NHS PH. |
| **Other information** | | | | |
| **Funding** | **22** | Give the source of funding and the role of the funders for the present study and, if applicable, for the original study on which the present article is based |  | Nil to declare. |
